# Correction: G82S RAGE polymorphism influences amyloid-RAGE interactions relevant in Alzheimer’s disease pathology

**DOI:** 10.1371/journal.pone.0248252

**Published:** 2021-03-04

**Authors:** Rani Cathrine Chellappa, Bincy Lukose, P. Rani

The first author’s name and initials appear incorrectly in the citation. The correct name is: Rani Cathrine Chellappa. The correct citation is: Chellappa RC, Lukose B, Rani P (2020) G82S RAGE polymorphism influences amyloid-RAGE interactions relevant in Alzheimer’s disease pathology. PLoS ONE 15(10): e0225487. https://doi.org/10.1371/journal.pone.0225487.
