# Supplementary figures and images for: G82S RAGE polymorphism influences amyloid-RAGE interactions relevant in Alzheimer’s disease pathology
Source: PLoS One. 2020 Oct 29;15(10):e0225487. doi: 10.1371/journal.pone.0225487 (PMC7595441; doi:10.1371/journal.pone.0225487)

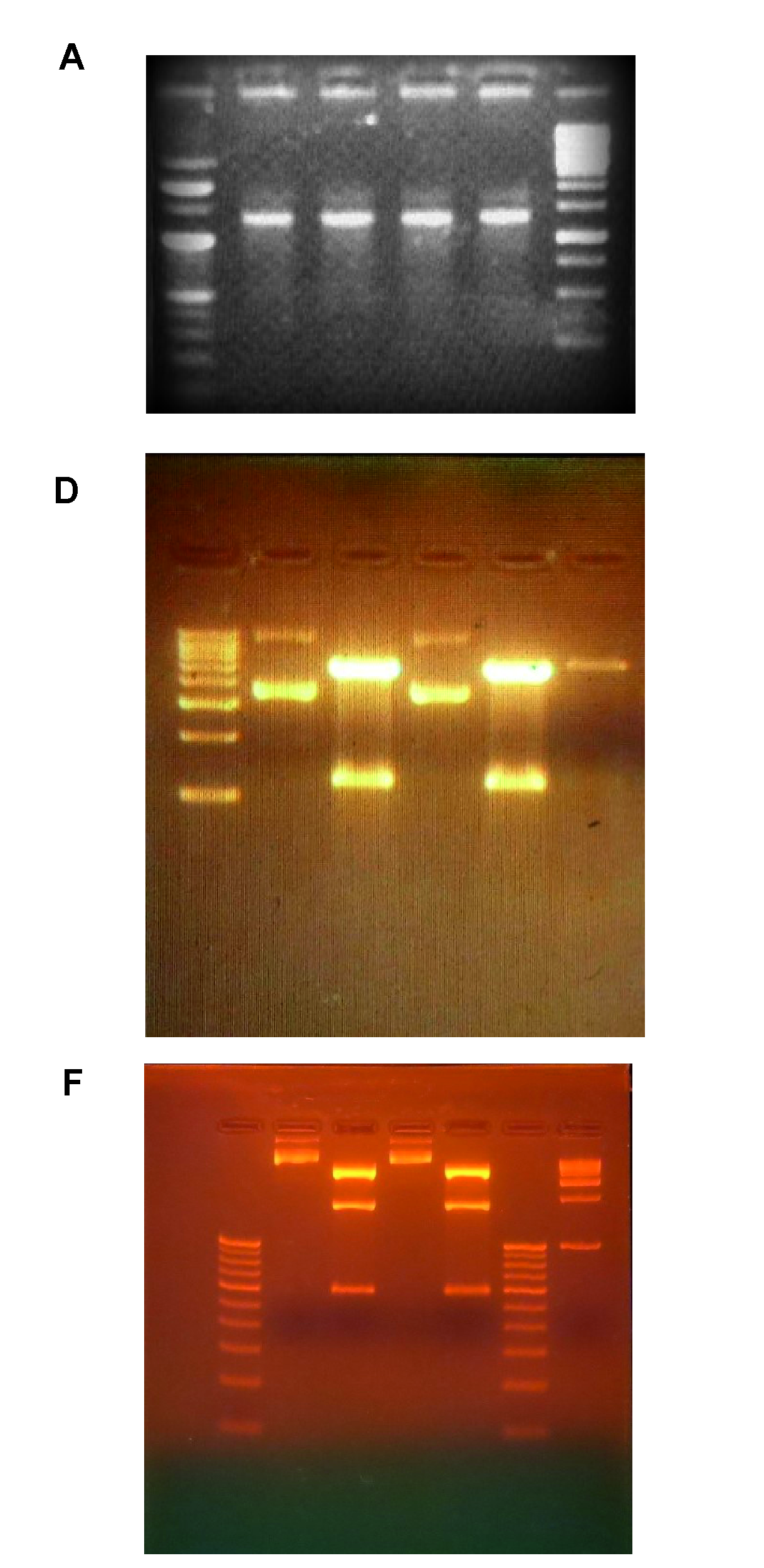

Supplement: S1 Fig — Restriction digestion of recombinant pcDNA3.1 construct with KpnI and EcoRI were electrophoresed in a 1% agarose gel (Fig 3D). To confirm orientation of cloned RAGE gene recombinant pcDNA3.1 construct was restricted with KpnI and SmaI and digested products were electrophoresed in a 2% agarose gel (Fig 3F). (TIF) [file pone.0225487.s001.tif]

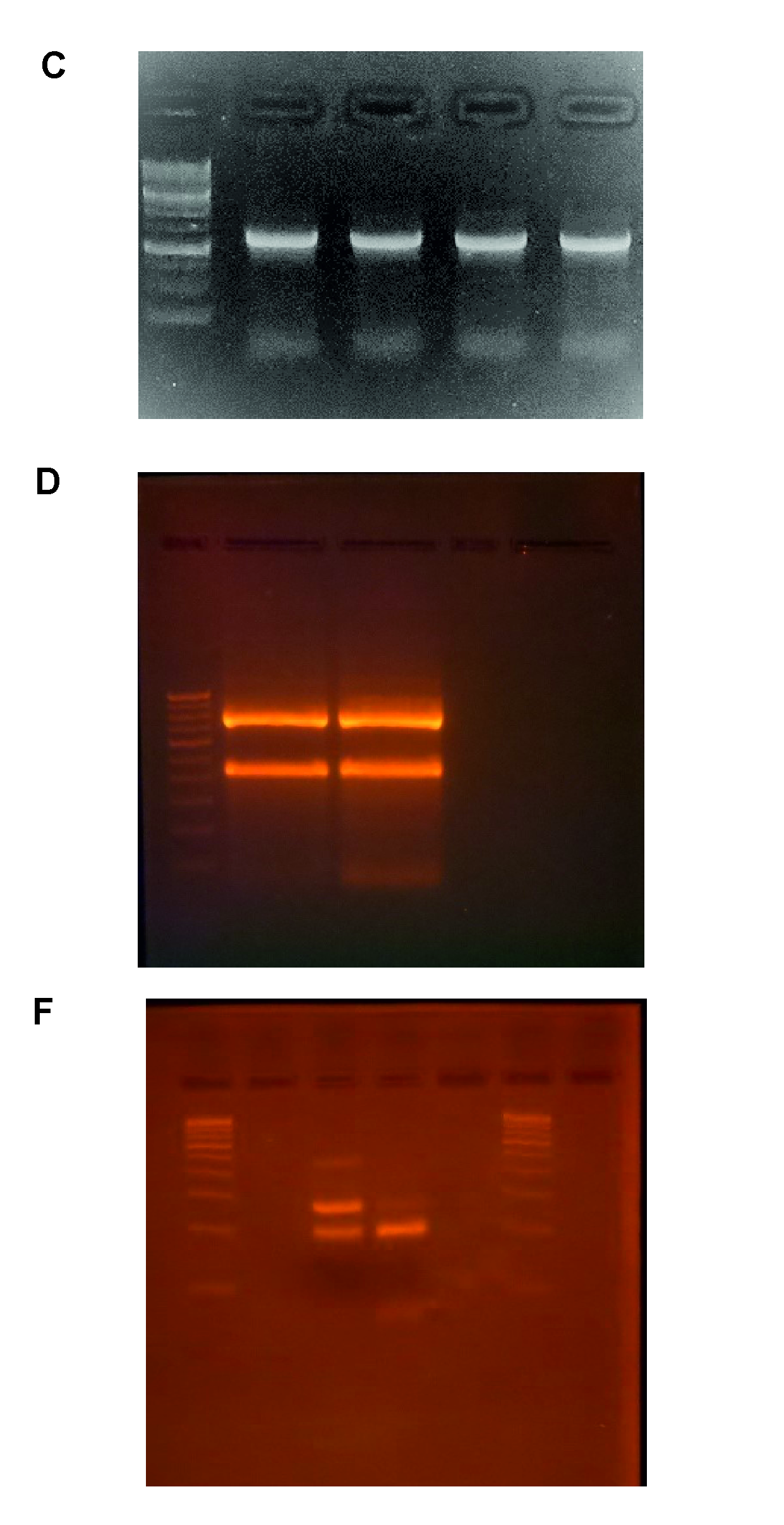

Supplement: S2 Fig — Restriction profile of AflIII digested fRAGE gene PCR product (Fig 4D). Restriction profiling of AluI digested WT and mutant RAGE gene (Fig 4E). (TIF) [file pone.0225487.s002.tif]

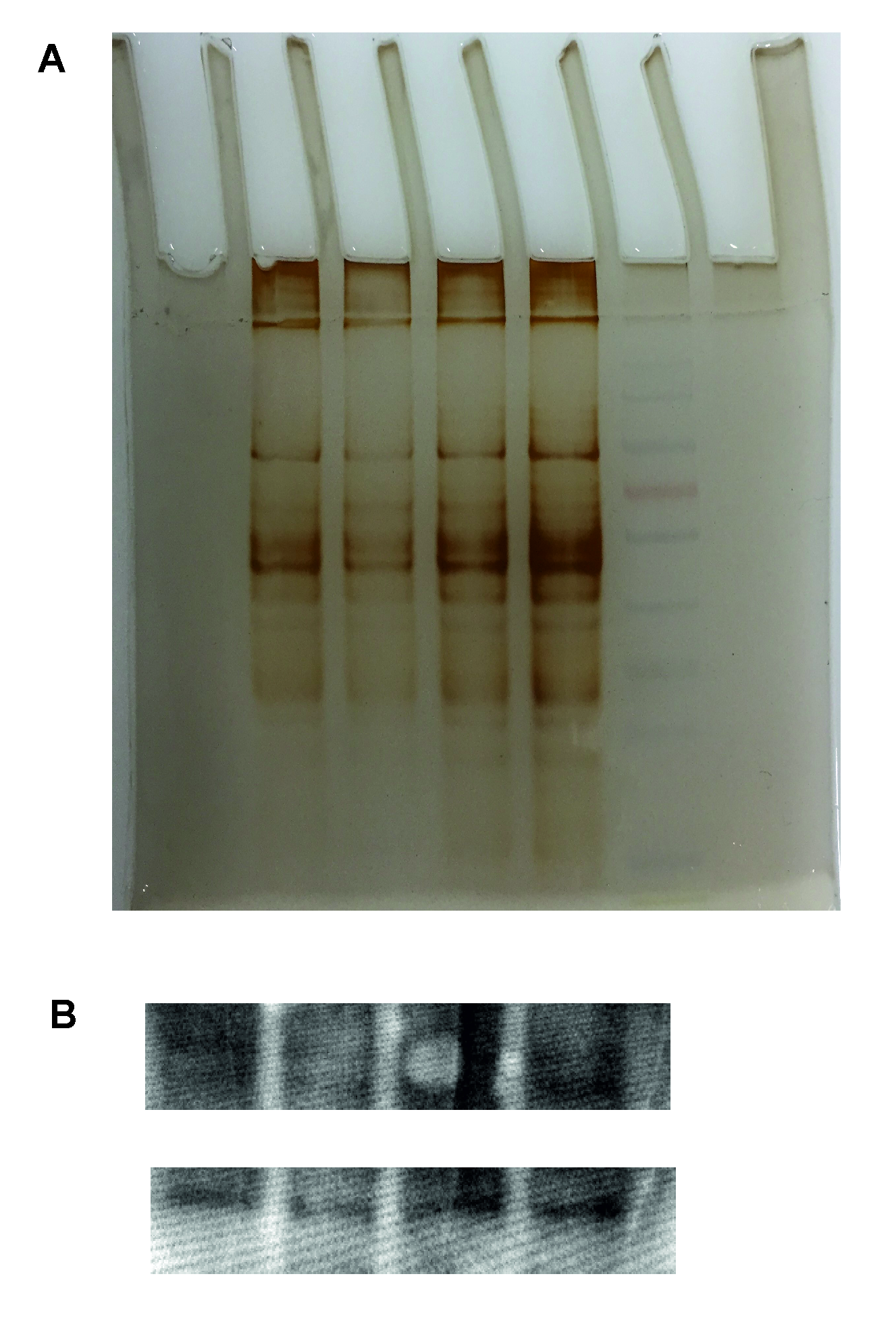

Supplement: S3 Fig — Western blot analysis of RAGE in transfected and non-transfected cells (Fig 5B). (TIF) [file pone.0225487.s003.tif]
